# The DNA Methylome of the Hyperthermoacidophilic Crenarchaeon *Sulfolobus acidocaldarius*

**DOI:** 10.3389/fmicb.2018.00137

**Published:** 2018-02-08

**Authors:** Mohea Couturier, Ann-Christin Lindås

**Affiliations:** Department of Molecular Biosciences, The Wenner-Gren Institute, Stockholm University, Stockholm, Sweden

**Keywords:** methylation, SMRT sequencing, cell cycle, transcription regulation, archaea, N^6^-methyl-adenine, N^4^-methyl-cytosine, 5-methyl-cytosine

## Abstract

DNA methylation is the most common epigenetic modification observed in the genomic DNA (gDNA) of prokaryotes and eukaryotes. Methylated nucleobases, N^6^-methyl-adenine (m6A), N^4^-methyl-cytosine (m4C), and 5-methyl-cytosine (m5C), detected on gDNA represent the discrimination mark between self and non-self DNA when they are part of restriction-modification systems in prokaryotes (Bacteria and Archaea). In addition, m5C in Eukaryotes and m6A in Bacteria play an important role in the regulation of key cellular processes. Although archaeal genomes present modified bases as in the two other domains of life, the significance of DNA methylations as regulatory mechanisms remains largely uncharacterized in Archaea. Here, we began by investigating the DNA methylome of *Sulfolobus acidocaldarius*. The strategy behind this initial study entailed the use of combined digestion assays, dot blots, and genome resequencing, which utilizes specific restriction enzymes, antibodies specifically raised against m6A and m5C and single-molecule real-time (SMRT) sequencing, respectively, to identify DNA methylations occurring in exponentially growing cells. The previously identified restriction-modification system, specific of *S. acidocaldarius*, was confirmed by digestion assay and SMRT sequencing while, the presence of m6A was revealed by dot blot and identified on the characteristic Dam motif by SMRT sequencing. No m5C was detected by dot blot under the conditions tested. Furthermore, by comparing the distribution of both detected methylations along the genome and, by analyzing DNA methylation profiles in synchronized cells, we investigated in which cellular pathways, in particular the cell cycle, this m6A methylation could be a key player. The analysis of sequencing data rejected a role for m6A methylation in another defense system and also raised new questions about a potential involvement of this modification in the regulation of other biological functions in *S. acidocaldarius*.

## Introduction

DNA methylations are one of the most well-known epigenetic modifications. The addition of a methyl group on the exogenous nitrogen of the adenine or cytosine forming N^6^-methyl-adenine (m6A) or N^4^-methyl-cytosine (m4C) modifications, respectively, or directly on the endogenous carbon leading to 5-methyl-cytosine (m5C) modification is carried out by DNA methyltransferases (DNA MTases) (Jeltsch, 2002). The search for strain-specific restriction endonuclease (REase) and/or DNA MTase activities was initiated a long time ago in the three domains of life—Bacteria, Archaea, and Eukaryotes—by performing digestion assays (Ehrlich et al., 1985; Bestor et al., 1988; Cano et al., 1988; Vogelsangwenke and Oesterhelt, 1988; Seeber et al., 1990). Digestion-based methods imply the use of methylation sensitive REases and detection of methylations only in a specific context. Today, the development of new sequencing technologies such as the single-molecule real-time (SMRT) sequencing, as an example, allows for identification of and direct mapping of the methylated motifs along the genome of an organism. This technology, based on the speed of incorporation of each nucleotides with kinetic signatures specific for each nucleobase modification (Flusberg et al., 2010; Clark et al., 2012), has identified the genomic distribution pattern of m6A in bacteria (Fang et al., 2012; Murray et al., 2012; Kozdon et al., 2013) and, more recently, in multicellular eukaryotes (Greer et al., 2015; Zhang et al., 2015) as well as in one archaeal strain (Ouellette et al., 2015). These covalent modifications of nucleobases are widespread in the three domains of life and participate in different cellular processes.

Three DNA methylations, m6A, m4C, and m5C, are known to be present in the genome of prokaryotes (Bacteria and Archaea) and were described as part of restriction-modification systems allowing discrimination between self and non-self DNA (Roberts et al., 2010, 2015). The recognition site on the host DNA is modified by a DNA MTase as a mark for protection from degradation while unmethylated recognition site from invading DNA is recognized and cleaved by the cognate REase. No defense system like this has yet been described in eukaryotes but it has been well-documented in bacteria as for example *Escherichia coli* (Arber and Dussoix, 1962; Casadesus and Low, 2006) and, with the increase of the number of archaeal genomes available, many restriction-modification systems have been identified (Roberts et al., 2010, 2015). However, few studies have characterized these systems in Archaea. The restriction-modification systems PabI/M.PabI present in *Pyrococcus abyssi* (Ishikawa et al., 2005; Watanabe et al., 2006) and SuaI/M.SuaI present in *Sulfolobus acidocaldarius* DSM639 (Prangishvili et al., 1985; Grogan, 2003) are the most well-described. On the same principle as in bacteria, archaeal DNA MTases, M.PabI, and M.SuaI, methylate recognition sites 5′-GT^m6^AC-3′ or 5′-GG^m4^CC-3′, respectively, which protect them from cleavage mediated by the corresponding REase. Therefore, m6A, m4C, and m5C modifications found in genomic DNA (gDNA) of prokaryotes and involved in restriction–modification systems are important to preserve the genome integrity of the cells. It is not known, however, whether DNA methylation has other functions in Archaea, but DNA MTases and by extension, DNA methylations, have been shown to be involved in regulation of gene expression and embryonic development in eukaryotes along with initiation of DNA replication and maintenance of genome integrity in bacteria. These “orphans” or solitary DNA MTases are not associated with a cognate REase.

In eukaryotes, mainly in mammals, m5C methylation is the most represented modification. It is tissue specific and its maintenance is the result of the action of three different DNA MTases, DNMT1, DNMT3A, and DNMT3B (Smith and Meissner, 2013). This DNA methylation is restricted to characteristic sites, named CpG islands, located at promoter regions of housekeeping genes and genes regulating development (Smith and Meissner, 2013). It has been shown, in cancer cells for example, that hypermethylation of CpG islands present in promoters of tumor suppressor leads to inhibition of their transcription (Esteller, 2005). Since m5C is widely distributed in mammals and is involved in human disease, this methylation has been the center of most epigenetic studies (Esteller, 2005; Smith and Meissner, 2013). However, an additional DNA methylation is also present in eukaryotic cells. Indeed, adenine DNA methylation has been detected on DNA in unicellular eukaryotes such as the alga *Chlamydomonas reinhardtii*, in plants and in mosquitos (Hattman et al., 1978; Ratel et al., 2006). More recently, this methylation was also discovered in DNA extracted from multicellular eukaryotes *Drosophila melanogaster* and *Caenorhabditis elegans* (Greer et al., 2015; Zhang et al., 2015). Even if the biological function of this DNA methylation remains unclear in eukaryotes, it has been proposed that it could play a role in transcription in *C. reinhardtii* (Fu et al., 2015) as well as being part of cross talk with histone methylations in *C. elegans* (Greer et al., 2015). This m6A modification might be considered as a new epigenetic mark for eukaryotes (Heyn and Esteller, 2015; Luo et al., 2015).

In bacteria, m6A methylation is the main DNA methylation detected and DNA adenine methyltransferase (Dam) and cell cycle-regulated methyltransferase (CcrM) present in Gamma-proteobacteria and Alpha-proteobacteria, respectively, are the best characterized examples of bacterial solitary DNA MTases. Although both MTases perform the same type of methylation, they exhibit different properties. The 5′-GATC-3′ recognition sequences are the targeted sequences of Dam while the 5′-GANTC-3′ sites (N being any nucleotide) are methylated by CcrM (Marinus and Morris, 1973; Geier and Modrich, 1979; Zweiger et al., 1994). In addition, Dam recognizes either hemi- or unmethylated recognition sequences, is processive and is not essential in enteric bacteria while CcrM has a preference for hemimethylated recognition sequences, is not processive and is essential. Studies revealed an involvement of adenine DNA methylation in regulation of the expression of genes involved in regulation of cellular processes such as the cell cycle for CcrM (Stephens et al., 1996), DNA replication along with pleiotropic roles for Dam (Wion and Casadesus, 2006). Finally, CcrM is expressed at specific stage of the cell cycle whereas Dam displays a constitutively expression in the cell.

Studies performed both in eukaryotes and bacteria not only highlight that DNA methylations are the result of dynamic processes but also their importance in the control of key cellular mechanisms, function which still remains unknown in Archaea. In this study, *S. acidocaldarius* strain DSM639 was selected as a suitable model archaeon to study the involvement of DNA methylations in regulatory mechanisms, cell cycle control in particular. Indeed, this aerobic hyperthermoacidophilic crenarchaeon (Brock et al., 1972) exhibits a restriction-modification system SuaI/M.SuaI (Prangishvili et al., 1985; Grogan, 2003) in contrary to other *Sulfolobales* (i.e., *Sulfolobus solfataricus, Sulfolobus islandicus*) and the sequencing of its genome in 2005 (Chen et al., 2005) identified genes encoding other potential MTases such as M.SuaII which was recently proposed to be a DNA cytosine-C5 MTase (Blow et al., 2016). Also, the cell cycle has been intensively studied in this microorganism (Lindås and Bernander, 2013), due to its capacity to be synchronized (Lundgren et al., 2004; Duggin et al., 2008). Therefore, we combined different technologies to, first, characterize the DNA methylome of *S. acidocaldarius* in exponentially growing cells. The DNA methylation associated to the well-known restriction-modification system SuaI/M.SuaI, m4C, was identified validating the use of the SMRT sequencing. The presence of m6A and m5C modifications was investigated by dot blot followed by SMRT sequencing in case of antibody detection. No signal corresponding to m5C was observed while, for the first time in a *Sulfolobales*, we could show the presence of a new DNA methylation, m6A, in two different contexts along the gDNA. Second, comparing sequencing data for both methylations, m4C and m6A, it seemed that m6A is not associated to a restriction-modification system, therefore, a question is if it could be involved in the regulation of the cell cycle. To test this hypothesis, we monitored the methylation profiles of synchronized cells of *S. acidocaldarius* DSM639 and, if m6A has a role, its involvement could be indirect as deduced from the sequencing data.

## Materials and methods

### Culturing of *S. acidocaldarius* DSM639 and sampling

Culturing of *S. acidocaldarius* strain DSM639 was performed in long neck flasks containing Allen media supplemented with 0.2% tryptone as previously described (Grogan, 1989) and were placed under continuous shaking at 80°C. The cell density was monitored by measuring the optical density at 600 nm (OD_600nm_). Cultures were grown exponentially until OD_600nm_ reached 0.1 in order to prepare independently asynchronous and synchronous cultures. For the analysis of the DNA methylome of asynchronous cells, 3.4 × 10^9^ cells were collected at 4°C and 2,300 × g for 10 min at OD_600nm_ = 0.1. To study the variations of the DNA methylome during the cell cycle, exponentially growing cells were synchronized by the addition of 3 mM acetic acid as described in Lundgren et al. (2004) and samples (3.4 × 10^9^ cells) were collected at 0, 30, 60, 90, and 120 min after acetate release.

### DNA extraction, whole genome amplification (WGA), and methylated DNA production

Genomic DNA extractions from asynchronous and synchronous *S. acidocaldarius* cells and 2.0 × 10^7^ cells from the Human cell line H1299 were performed using the genomic-tip 100/G kit (Qiagen) following the manufacturer's recommendations. Dried pellets were resuspended in 0.22 μm filtered milliQ-water, the corresponding concentration and purity were measured using Nanodrop and then stored at −20°C. Cells from H1299 cell line were provided by Wojcik's group at Stockholm University, Sweden. Genomic DNA extracted from asynchronous *S. acidocaldarius* cells (10 ng) was amplified during 17 h at 30°C using the Repli-g mini kit (Qiagen) and used as negative control for the search of all DNA modifications. An aliquot (250 ng) of the whole genome amplification (WGA) sample was methylated for 1 h at 37°C by 0.002 Units of Dam methyltransferase in presence of 80 μM S-adenosylmethionine (New England BioLabs) in order to create the positive control for the detection of m6A methylations.

### Digestion assays

Genomic DNA isolated from asynchronous *S. acidocaldarius* cells was cleaved independently with a specific REase in order to decipher the presence or the absence of m4C or m6A: BsuRI, BamHI, MboI, and DpnI (Thermo Scientific). Briefly, digestions of 250 ng of gDNA by 2.5 Units of enzyme were performed for 30 min at 37°C and loaded onto a 0.8% agarose gel at room temperature in TAE buffer (40 mM Tris-acetate, 2 mM EDTA, pH 8.0), stained with ethidium bromide (2 μg mL^−1^) and digitalized under UV light.

### Detection of m6A and m5C on genomic DNA by dot blot

Genomic DNA extracted from exponentially growing cells, WGA and WGA modified by Dam methyltransferase samples were diluted to 100 ng μl^−1^ and denatured in 0.4 M NaOH and 10 mM EDTA pH 8.2 solution during 10 min at 95°C. Samples were placed on ice and 500 ng were loaded manually per dot on a wetted positively charged Nylon membrane (Roche). The membrane was briefly washed in SSC buffer (0.3 M NaCl, 0.03 M Sodium citrate pH 7.2), allowed to air dry and then blocked in 5% milk TBS (10 mM Tris-HCl pH 8.0, 150 mM NaCl) for 1 h at room temperature. Thereafter, the membrane was incubated for 1 h at room temperature with monoclonal primary antibodies raised either against m6A (1:2,500 dilution, C15200082; Diagenode) or m5C (1:600 dilution, C15200003; Diagenode) in 5% milk TBS, washed three times for 10 min with TBS supplemented with 0.1% Tween 20 (TBST buffer) and then probed with secondary antibodies (1:10,000 dilution, ThermoFisher Scientific) in 5% TBS for 1 h at room temperature. Three washes using TBST buffer were performed before applying ECL solution (SuperSignal^TM^ West Pico Chemiluminescent Substrate, ThermoFisher Scientific). The chemiluminescence and the methylene blue signals were captured using a CCD camera (ChemiDoc, BioRad) and the signals were analyzed with Image Lab (v 3.0.1) package.

### PacBio single-molecule real-time sequencing

The DNA methylomes of gDNA extracted from asynchronous and synchronous cells were obtained by using PacBio SMRT sequencing at SciLife, Uppsala, Sweden. DNA was sheared into 10 kb fragments using a Genemachines HydroShear Instrument (Digilab, Marlborough, MA, USA). SMRT-bells were constructed according to the manufacturer's instructions (Pacific Biosciences, Menlo Park, CA, USA). SMRT-bells longer than 7 kb were selected by BluePippin (Sage Science, Beverly, MA, USA) and sequenced on one SMRT-cell per sample using a Pacific Biosciences RSII sequencer according to the manufacturer's instructions with 4 h movie-time, yielding 221.78x coverage for asynchronous gDNA, 278.33; 289.31; 348.92; 333.91; and 328.97x coverage for synchronous gDNA extracted at T0, T30, T60, T90, and T120 min after release, respectively. DNA modification detection and motif analysis were performed using RS Modification and Motif Analysis.1 method available on PacBio SMRT analysis platform (v 2.3.0). The “motifs.xlsx” files are available in Supplementary Material.

## Results

### Study of the DNA methylome of *S. acidocaldarius* DSM639 combining multiple experimental approaches

#### Indirect evidence of the presence/absence of 5′-GG^m4^CC-3′ and 5′-G^m6^ATC-3′ by digestion assays

**Figure 1:**
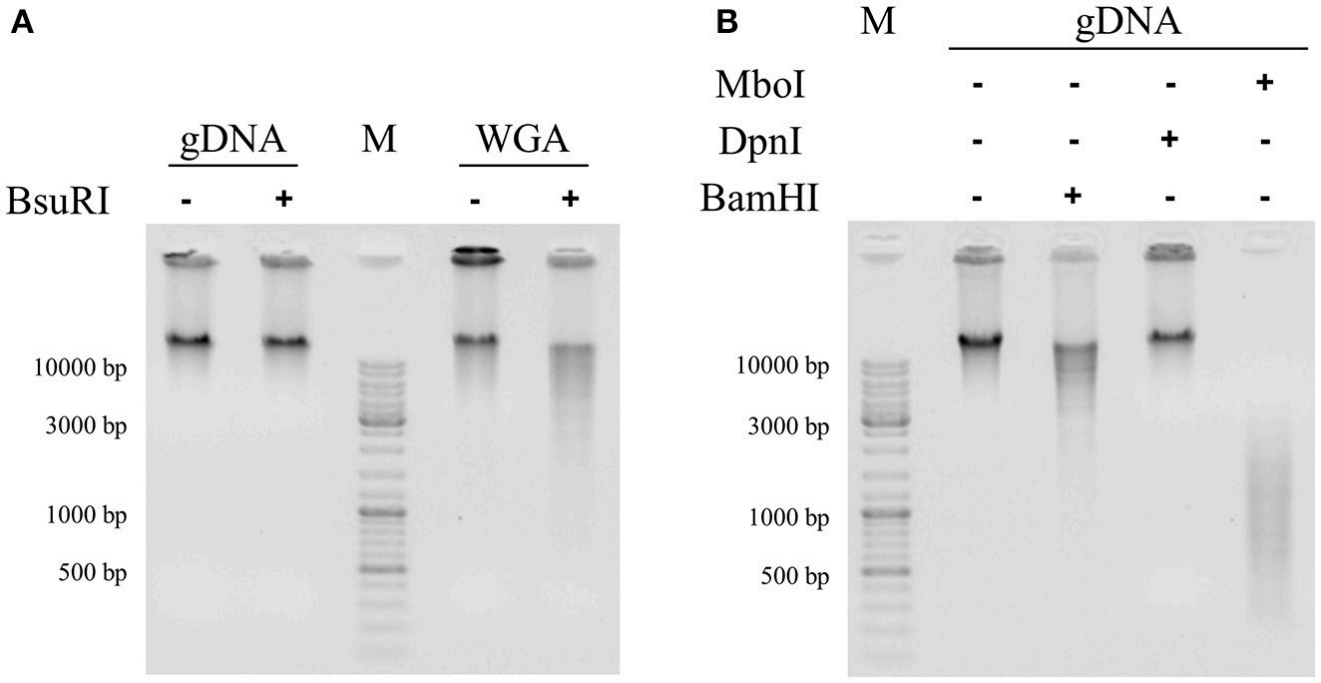
Identification of methylated recognition sequences in *S. acidocaldarius* genomic DNA by digestion assays. Restriction enzymes BsuRI **(A)** and BamHI, DpnI, and MboI **(B)** were used to highlight the presence/absence of methylated 5′-GGCC-3′ and 5′-GATC-3′ palindromes, respectively, in genomic DNA of *S. acidocaldarius*. Two types of substrates were digested: genomic DNA (gDNA) and a whole genome amplification of the genomic DNA (WGA). WGA is identical to gDNA in terms of nucleic sequence but it does not contain epigenetic marks (see material and methods). This negative control is only used in **(A)**. The addition of different restriction enzymes is symbolized by a positive sign “+” while reaction mix without restriction enzyme is represented by a negative sign “–”. Molecular size markers (GeneRuler DNA Ladder, Thermo Scientific) were loaded in both gels (M). Digestion patterns were obtained in 0.8% agarose gel stained with ethidium bromide and visualized under UV light.

Before investigating the potential link between the cell cycle and DNA methylation in *S. acidocaldarius*, the study of the DNA methylome was performed to identify DNA methylations. To do so, we first performed digestion assays by using specific restriction enzymes and gDNA extracted from exponentially growing cells as substrate. The presence of the 5′-GGCC-3′ motif, forming the recognition sequence characteristic of the restriction-modification system of *S. acidocaldarius*, was investigated by using the restriction enzyme BsuRI (Figure 1A) while that of the motif 5′-GATC-3′, part of the recognition sequence specific of Dam methylation, was highlighted independently by BamHI, DpnI, and MboI (Figure 1B).

In Figure 1A, two different substrates were used: gDNA and an amplification of the whole genome (WGA). The nucleic acid sequence of the latter substrate was identical to the gDNA but epigenetic marks were absent and, therefore, comparing digestion profiles of the sample (gDNA) and the negative control (WGA) by BsuRI allows us to check for the presence/absence of methylated cytosine (m4C) on the 5′-GGCC-3′ recognition sequence. In absence of BsuRI, both gDNA and WGA migrated to the same electrophoretic position (above 10 kbp) corresponding to 2.2 Mbp (Figure 1A). However, in presence of BsuRI, only WGA was digested since several bands were observed between 2.2 Mbp and 3 kbp while the gDNA still migrated to the same position (Figure 1A). This experiment clearly showed that the 5′-GGCC-3′ recognition sequences present on the gDNA were modified and these specific modifications protected it from digestion. The methylation associated with these recognition sequences was the addition of a methyl group on a cytosine (m4C) as described in previous studies (Prangishvili et al., 1985; Grogan, 2003).

To investigate the presence of both the 5′-GATC-3′ recognition sequences and the associated methylation on the adenine (m6A), three restriction enzymes were used: BamHI which recognizes 5′-GATC-3′ sequences in the context 5′-GGATCC-3′ independent of the methylation state, DpnI and MboI which both recognized 5′-GATC-3′ sequences but required either fully methylated or unmethylated palindromes, respectively, to cleave. In absence of restriction enzyme, gDNA migrated higher than 10 kbp corresponding to undigested gDNA (Figure 1B). In presence of each restriction enzymes, three different digestion profiles were observed: digestion by BamHI leading to long digestion products from 2.2 Mbp to 3 kbp, no digestion by DpnI and digestion by MboI resulting in shorter fragments from 2 kbp to 300 bp (Figure 1B). The fact that gDNA extracted from exponentially growing cells was not digested by DpnI but was digested by MboI indicated that 5′-GATC-3′ recognition sequences were present in the gDNA but they were not fully methylated. The two different digestion profiles using BamHI and MboI suggested that the 5′-GATC-3′ recognition sequences were present in, at least, two contexts where one, 5′-GGATCC-3′, appeared to be less abundant (Figure 1B).

#### Detection of DNA methylations using DNA modification-specific antibodies

**Figure 2:**
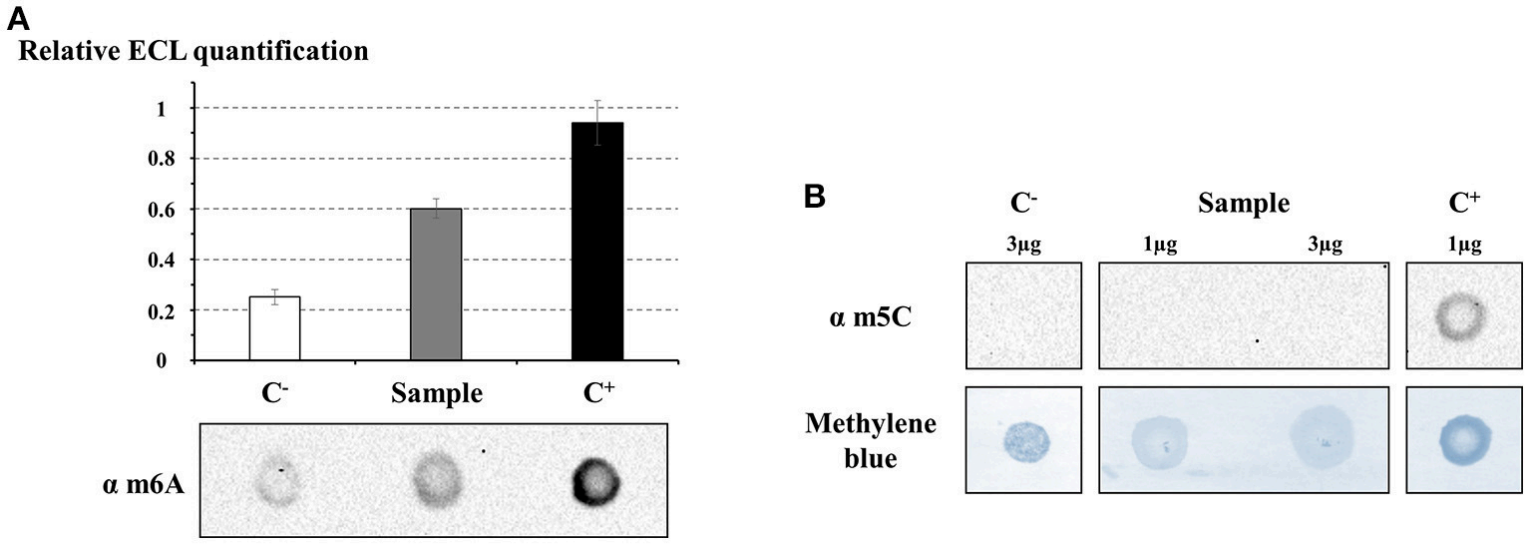
Direct detection of m6A and m5C methylations on genomic DNA of *S. acidocaldarius* using specific antibodies. The presence of m6A **(A)** and m5C **(B)** methylations were investigated by dot blot experiments. Genomic DNA extracted from *S. acidocaldarius* (gDNA) was added together with a negative control, C^−^ (WGA), and a positive control, C^+^ (WGA methylated *in vitro* by Dam methyltransferase), in **(A)** and genomic DNA extracted from H1299 cells in **(B)**. A quantification of the ECL signal corresponding to the detection of m6A, including error bars indicating the standard deviation, is presented in association with representative dot blot results of three independent experiments below **(A)**. The dot blot shown in **(B)** is representative of results obtained for two independent experiments. Methylene blue staining of DNA was considered as a loading control.

In order to investigate the presence of m6A and m5C on the gDNA of *S. acidocaldarius*, dot blot experiments using primary antibodies specifically raised against the corresponding modified base were performed (Figure 2). Both ECL and corresponding methylene blue signal of three independent experiments were quantified separately. Thereafter, the ECL signals were normalized against the methylene blue signals which were used as loading controls (Figure 2A). The DNA methylation m6A was detected in all three loaded samples but with different signal intensities (Figure 2A). Indeed, the signal for the gDNA was 1.5 times lower than the signal obtained using the positive control (fully methylated) but approximately 3 times stronger than the signal obtained using the negative control (Figure 2A). Consequently, this experiment highlighted that the gDNA of *S. acidocaldarius* contains m6A but not at all positions.

The same strategy was used to investigate the presence of m5C on gDNA extracted from *S. acidocaldarius* with the positive control being the gDNA extracted from the Human cell line H1299 (ATCC: https://www.atcc.org/). Antibodies specifically raised against m5C recognized the DNA methylation in the positive control sample (C^+^) while no signal was visible for the negative control (C^−^: WGA) even if the amount loaded was higher than for the positive control (Figure 2B; 3 vs. 1 μg, respectively). Two quantities of gDNA extracted from *S. acidocaldarius* (gDNA) were added onto the membrane to increase the probability to detect m5C (Figure 2B). However, no signal was observed for this sample for any of the quantities tested indicating that the number of m5C modifications, if present in the genome of *S. acidocaldarius*, was under the detection threshold of the antibodies.

#### Analysis of the DNA methylome of *S. acidocaldarius* DSM639 using SMRT sequencing

**Figure 3:**
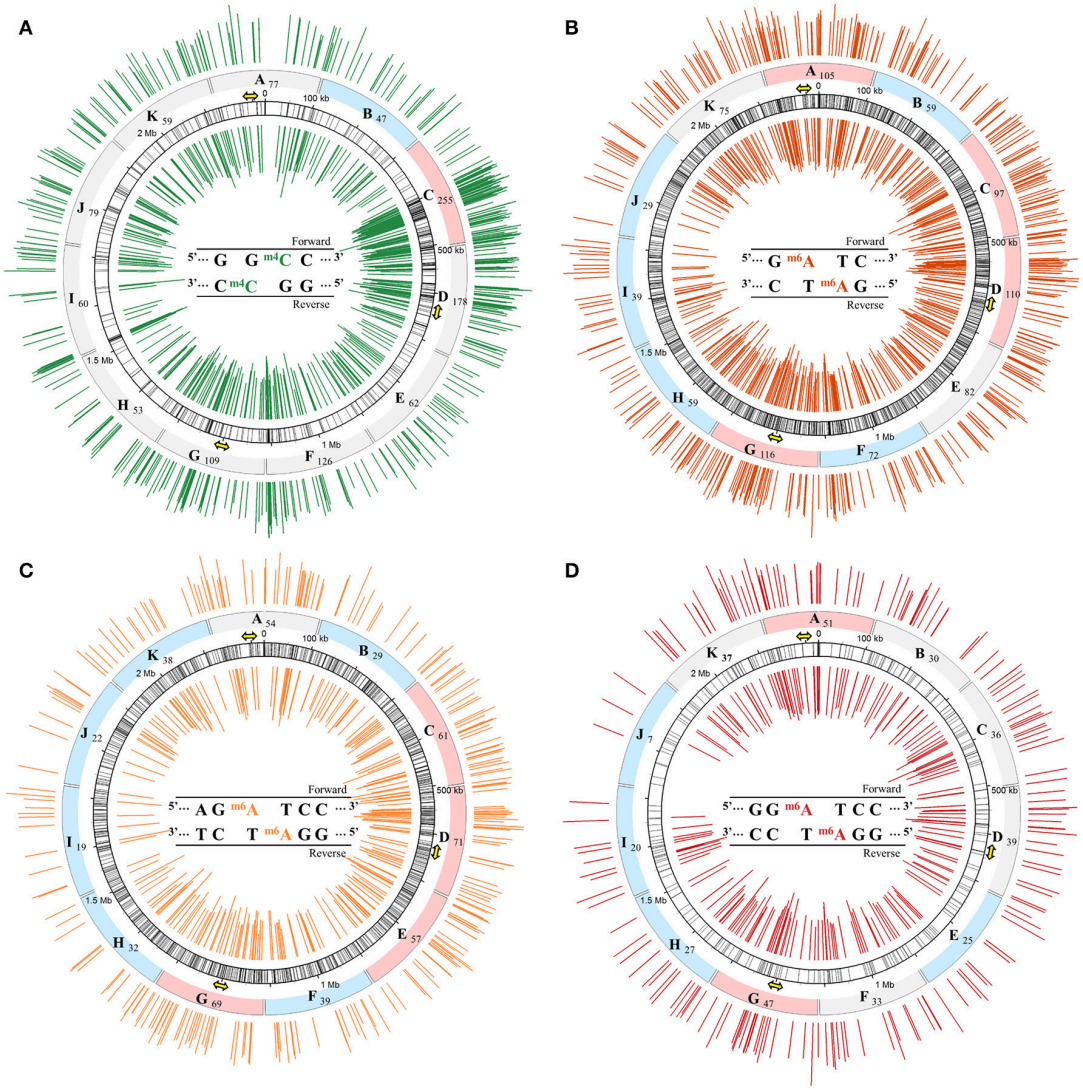
Distribution of the m4C and m6A methylations associated with their corresponding recognition sequences along the genomic DNA of *S. acidocaldarius*. Positions of each motifs: 5′-GGCC- 3′ **(A)**, 5′-GATC-3′ **(B)**, 5′-AGATCC-3′ **(C)**, and 5′-GGATCC-3′ **(D)** are reported on the entire double stranded genome represented by the numbered track (black circle). By base pairing, these motifs form specific recognition sequences symbolized by black lines. IPD ratio data corresponding only to m4C **(A)** and m6A **(B–D)** are reported on both strands over the genome with the inner and outer circle representing the reverse and forward DNA strands, respectively. The methylated base is colored in each specific recognition sequences in the center of each circle. Position of the three origins of replication are symbolized by the yellow arrows. The genome was divided into 11 sections from A to K and number of methylated motif counted in each portion is reported. Portions are filled in blue, red, or gray indicating whether they are hypo-, hypermethylated, or in the 95% confidence interval, respectively. Four plots were designed using the software Circos (Krzywinski et al., 2009).

To confirm the presence of DNA methylations m4C and m6A and map their genomic distributions, SMRT sequencing was applied to gDNA extracted from exponential growing cells (Supplementary Data Sheet 1). As expected, the 5′-GGCC-3′ recognition sequences belonging to the restriction-modification system of *S. acidocaldarius* were detected along the genome and the associated m4C methylation was also identified. In total and on both strands, 1,140 5′-GGCC-3′ motifs were counted forming 570 recognition sequences (Table 1). Almost all, 97% of the 5′-GGCC-3′ motifs were methylated (Table 1). Since SMRT sequencing provided positions of each motifs and associated modifications, we could report them along the gDNA (Figure 3) and identify the methylation state of the 5′-GGCC-3′ recognition sequences (Figure 3A). After calculations based on the percentage of methylated 5′-GGCC-3′ motifs and their distributions along the genome, we found that 94% of the 5′-GGCC-3′ motifs were engaged in fully methylated recognition sequences while only 6% in hemimethylated state.

**Table 1:** Motifs and associated methylations present along the genomic DNA of *S. acidocaldarius*.

**Motif**	**Nber of motif in the genome**	**Nber of recognition sequences in the genome**	**Type of methylation**	**% of methylated motif**
5′-GG**C**C-3′	1,140	570 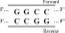	m4C	97
5′-AG**A**TCC-3′	1,022	1,022		
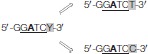	1,022	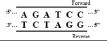	m6A	24
	578	289 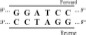	m6A	60.9
5′-G**A**TC-3′	2,622	1,311 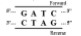	m6A	32.1

*The SMRT sequencing revealed the presence of three motifs: 5′-GG**C**C-3′, 5′-AG***A***TCC-3,′ and 5′-GG***A***TCY-3′ with “Y” being a pyrimidine (“T” or “C”). The two latter motifs contain 5′-G***A***TC-3′ as core motif (underlined in both) which can be studied as such. Modified nucleobases, for each motif, are written in bold. The number of motifs in the whole genome of S. acidocaldarius, the number of corresponding recognition sequences formed in the genome, the type of methylation and the percentage of methylated motifs are given in this table*.

The genome was divided into 11 sections of 202,360 bp (from A to K in Figure 3) and the number of methylated 5′-GGCC-3′ recognition sequences was reported for each of them in order to identify hyper- and hypomethylated chromosome regions (Figure 3). The Shapiro test was applied to determine the distribution of methylated 5′-GGCC-3′ recognition sequences: W = 0.93 and *p* = 0.007 meaning methylated 5′-GGCC-3′ recognition sequences were abnormally distributed. Thereafter, the 95% confidence interval (95%CI) was calculated using the median of the number of methylated sites (median = 77) and taking into account the ranking: 77[95CI% 53–178]. Following this, each portions presenting a number of methylated 5′-GGCC-3′ recognition sequences higher or lower than the 95% confidence interval was considered as hypermethylated or hypomethylated, respectively. According to Figure 3A, portion C was considered as hypermethylated while portion B was considered as hypomethylated. Portions A, D, and G containing one origin of replication each, symbolized by a yellow arrow (oriC2: 2,220 kb; oriC1: 630 kb; and oriC3: 1,200 kb; Lundgren et al., 2004; Duggin et al., 2008), and none of them were considered as hyper- or hypomethylated (Figure 3A).

The analysis of the sequencing data showed that 89.6% of the methylated 5′-GGCC-3′ recognition sequences were in open reading frames suggesting that most of the coding sequences were protected from SuaI cleavage. Furthermore, we investigated whether the distribution of 5′-GGCC-3′ recognition sequences in coding sequences was linked to a specific archaeal cluster of orthologous genes [arCOG (Makarova et al., 2007)]. We observed that methylated 5′-GGCC-3′ recognition sequences were not only present in roughly all arCOGs but were also found in genes encoding tRNA and rRNA (Table 2). Only two arCOGs, N and U (N: Cell motility and U: Intracellular trafficking and secretion), did not contain any 5′-GGCC-3′ recognition sequences (Table 2).

**Table 2:** Distribution of detected motifs in coding sequences and estimation of the percentage of the associated methylation.

	5^**′**^-GG**C**C-3^**′**^		5^**′**^-G**A**TC-3,^**′**^		5^**′**^-AG**A**TCC-3^**′**^		5^**′**^-GG**A**TCC-3^**′**^	
	**Norm. I**	**Norm. II**	**Norm. I**	**Norm. II**	**Norm. I**	**Norm. II**	**Norm. I**	**Norm. II**
**arCOG**								
**METABOLISM**								
C: Energy production	18.2	90.6	47.6	29.5	35	22.1	12.6	53.8
E: Amino acid metabolism and transport	12.4	98.1	59.5	41.3	43.8	30.6	15.7	78.3
F: Nucleotide metabolism and transport	1.8	100	45.5	40	36.4	34.8	9.1	57.1
G: Carbohydrate metabolism and transport	23.5	88.2	49	34.2	35.3	27.6	13.7	55.6
H: Coenzyme metabolism and transport	7.7	94.4	37.4	30.9	30.8	20.3	6.6	70
I: Lipid metabolism and transport	30.9	94.2	51.5	36.1	33.8	28.6	17.6	50
P: Inorganic ion metabolism and transport	10.9	80	41.3	25.9	34.8	20.5	6.5	50
Q: Secondary metabolites biosynthesis, transport and catabolism	13.8	87.5	48.3	30.6	37.9	14.3	10.3	87.5
**INFORMATION STORAGE AND PROCESSING**								
J: Translation	17.3	100	33.9	43.5	28.6	39.1	5.4	19
K: Transcription	7	96.9	22.5	37.8	19.4	30.6	3.1	9
L: Replication, recombination and repair	16.7	100	39.7	36.7	29.5	24.2	10.3	70.8
**CELLULAR PROCESSES AND SIGNALING**								
D: Cell cycle control	20	100	40	30	40	30	0	0
M: Cell wall, membrane and envelope biogenesis	8.3	90	47.9	30.6	31.3	17.5	16.7	54.5
N: Cell motility	0	0	50	25	25	0	25	50
O: Post-translational modification, protein turnover, chaperones	14.3	100	44.4	46.2	36.5	35	7.9	83.3
T: Signal transduction mechanisms	8.3	100	25	50	16.7	50	8.3	50
U: Intracellular trafficking and secretion	0	0	25	41.7	25	41.7	0	0
V: Defense mechanisms	7.8	100	20.8	19	18.2	13.9	2.6	50
X: Mobilome	6.1	100	21.2	14.3	15.2	0	6.1	50
**POORLY CHARACTERIZED**								
R: General functional prediction only	7.8	91.7	44.2	26.5	34.1	17.1	10.1	62.5
S: Function unknown	10.7	98.8	28.1	28.2	23.3	20.3	4.8	59.7

**Genes encoding tRNA**	81.6	100	8.2	12.5	8.2	12.5	0	0
**Genes encoding rRNA**	100	100	50	10	50	10	0	0

*The percentage of genes containing the recognition sequence, 5′-GG**C**C-3′, 5′-AGATCC-3′, 5′-GGATCC-3,′ and/or the core motif 5′-GATC-3′ determined in Table 1, was calculated by counting the number of the corresponding motif in each genes. Then, this number was normalized using the total number of genes present either in each arCOG or in genes encoding tRNA or rRNA (Norm. I). To go further, the percentage of methylated motif was estimated by sorting methylated motif and normalizing by the total number of motif detected (Norm. II)*.

The SMRT sequencing analysis confirmed the presence of m6A DNA methylation on the gDNA of *S. acidocaldarius*. Addition of a methyl group on the adenine, observed for the first time in this study by dot blot experiments (Figure 2A), was identified by SMRT sequencing on two motifs, 5′-AGATCC-3′ and 5′-GGATCY-3′ (with “Y” being a pyrimidine), both containing the core motif 5′-GATC-3′ (Table 1). By analyzing different reads obtained by the sequencing, the “Y” was identified as a “T” (5′-GGATCT-3′) which complemented 1,022 5′-AGATCC-3′ sub-motifs forming 1,022 5′-AGATCC-3′ recognition sequences or a “C” (5′-GGATCC-3′) which could interact with each other resulting in 289 5′-GGATCC-3′ recognition sequences (Table 1). The percentage of m6A for these two recognition sequences was calculated to 24 and 60.9%, respectively (Table 1). They can be considered independently or together by following the 5′-GATC-3′core motif (Table 1). There were 2,622 5′-GATC-3′ motifs on the genome forming 1,311 recognition sequences and the percentage of methylated 5′-GATC-3′ recognition sequences was 32.1% (Table 1). The distribution of these three recognition sequences associated with their methylations is reported in Figures 3B–D.

In the same way as for m4C, we identified portions of the genome which were considered as hyper- or hypomethylated (m6A) on 5′-AGATCC-3′ and 5′-GGATCC-3′ recognition sequences (Figures 3C,D). The Shapiro test was applied on both methylated recognition sequences where W = 0.93 and *p* = 0.42 and W = 0.98 and *p* = 0.95, respectively, indicating a normally distributed methylation pattern. By consequence, the 95% confidence interval (95%CI) of both recognition sequences was calculated considering the mean of the number of methylations (${\text{mean}}_{5^{\prime }-\text{AGATCC}-3^{\prime }}$ = 45 and ${\text{mean}}_{5^{\prime }-\text{GGATCC}-3^{\prime }}$ = 32): 45[95CI% 43–55] or 32[95CI% 30–42] for 5′-AGATCC-3′ and 5′-GGATCC-3′ recognition sequences, respectively. Each portions presenting a number of methylated recognition sequences higher or lower than the corresponding 95% confidence interval were considered as hyper- or hypomethylated. In Figure 3C, portions B, F, H, I, J, and K were hypomethylated and portions C, D, E and G were hypermethylated while in Figure 3D, portions E, H, I, and J were hypomethylated and portions A, and G were hypermethylated. For the m6A methylation, two of the three origins of replication were found in hypermethylated portions, but only one, oriC3, was common for both contexts.

The combined analysis of these representations of the SMRT sequencing data showed that 78% of 5′-GATC-3′ recognition sequences were part of the 5′-AGATCC-3′ context while 22% were part of the 5′-GGATCC-3′ context (Figures 3C,D). The two recognition sequences 5′-AGATCC-3′ and 5′-GGATCC-3′ were different in terms of numbers along the genome but also in their methylation state. Indeed, taking the total number of each recognition sequences, the corresponding percentage of methylation and their distribution along the genome, 12.9% of 5′-AGATCC-3′ recognition sequences against 42.9% of 5′-GGATCC-3′ recognition sequences were fully methylated (Figure 3C compared to Figure 3D).

Since 92.7% of the 5′-GATC-3′ recognition sequences were present in coding sequences and part of the two sequences 5′-AGATCC-3′ and 5′-GGATCC-3′, we could deduce these two recognition sequences localize in coding sequences as well. Table 2 provides an overview of the distribution of the 5′-GATC-3′, 5′-AGATCC-3′, and 5′-GGATCC-3′ recognition sequences in different arCOGs. Although 5′-GATC-3′ recognition sequences were present in genes belonging to each arCOGs, genes of clusters E, I, and N (E: Amino acid metabolism and transport, I: Lipid metabolism and transport, and N: Cell motility) and genes encoding rRNA exhibited more of these recognition sequences. However, these genes were not the most highly methylated. Indeed, the percentage of methylated recognition sequences per arCOG was high compared to the number of recognition sequences in clusters J, K, O, T, and U (J: Translation, K: Transcription, O: Post-translational, protein turnover, chaperones, and U: Intracellular trafficking and secretion) and genes encoding tRNA (Table 2, 5′-GATC-3′, Norm. II). Concerning the two other recognition sequences, they showed slight differences (Table 2, 5′-AGATCC-3′ and 5′-GGATCC-3′). First, 5′-AGATCC-3′ recognition sequences were not only present in all arCOGs but also in genes encoding either tRNA or rRNA while 5′-GGATCC-3′ recognition sequences were absent from genes belonging to clusters D and U (D: Cell cycle and U: Intracellular trafficking and secretion) and genes encoding either tRNA or rRNA (Table 2, 5′-AGATCC-3′ and 5′-GGATCC-3′, Norm. I). Second, the percentage of methylated recognition sequences found in the column Norm. II of the Table 2 was always higher for 5′-GGATCC-3′ recognition sequences than for 5′-AGATCC-3′ recognition sequences and could be zero for genes containing the latter. For example, this was observed for genes belonging to the arCOGs N and X (N: Cell motility and X: Mobilome) which contained the 5′-AGATCC-3′ recognition sequences but they were not methylated (Table 2, motif AGATCC).

### Adenine DNA methylation profiles during the cell cycle of *S. acidocaldarius* DSM639

Once the use of SMRT sequencing was validated for the study of the DNA methylome, it was possible to investigate whether a link existed between DNA methylations and the regulation of the cell cycle in *S. acidocaldarius*. Cells were synchronized by adding acetic acid as described in Lundgren et al. (2004) and collected at different time points of the cell cycle: 0, 30, 60, 90, and 120 min after the release of the acetic acid. For each time point, gDNA was extracted and analyzed by SMRT sequencing (Supplementary Data Sheets 2–6). Motifs and methylation types revealed by the analysis of the gDNA extracted from synchronous cells were found for each time point and the results are reported in Table 3. The 5′-GGCC-3′ recognition sequences were mainly found methylated and the percentage was roughly constant (around 99%) during the cell cycle (Table 3). Once again, the methylation state of the 5′-GATC-3′ core motif was different depending on the two contexts. Indeed, the percentage of methylation of the adenine was higher in the context 5′-GGATCC-3′ than in the context 5′-AGATCC-3′ by approximately a factor of 2.5. However, the percentage of m6A in both contexts followed the same variations. During the first 30 min the level of m6A was roughly constant, where after it dropped at T60′ (G1), slightly increased at T90′ (S) and decreased again at T120′ (G2) (Table 3). Even if T0 and T120 min were described as being G2 stage, the level of m6A for both contexts was different.

**Table 3:** Variations of percentage of methylated motifs 5′-GG^m4^CC-3′ and 5′-G^m6^ATC-3′ during the cell cycle of *S. acidocaldarius*.

		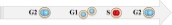				
		**T0 (%)**	**T30′ (%)**	**T60′ (%)**	**T90′ (%)**	**T120′ (%)**
	5′-GG^m4^**C**C-3′	98.4	98.8	98.8	98.7	99.2
5′-G^m6^**A**TC-3′	5′-AG^m6^**A**TCC-3′	24.94	24.56	17.61	21.28	18.74
	5′-GG^m6^**A**TCC-3′	61.76	66.26	51.76	55.88	50.52

*Motif 5′-GATC-3′ considered as a core motif is directly divided into two contexts: 5′-AGATCC-3′ and 5′-GGATCC-3′. Since the percentage of methylation is considered, the methylated base is written in bold in the corresponding motif. Five time points collected after acetic acid released: T0, T30′, T60′, T90′, T120′ are associated with their respective state of the cell cycle. These states are reported in the time line above with non-replicating DNA in blue and replicating DNA in red*.

**Figure 4:**
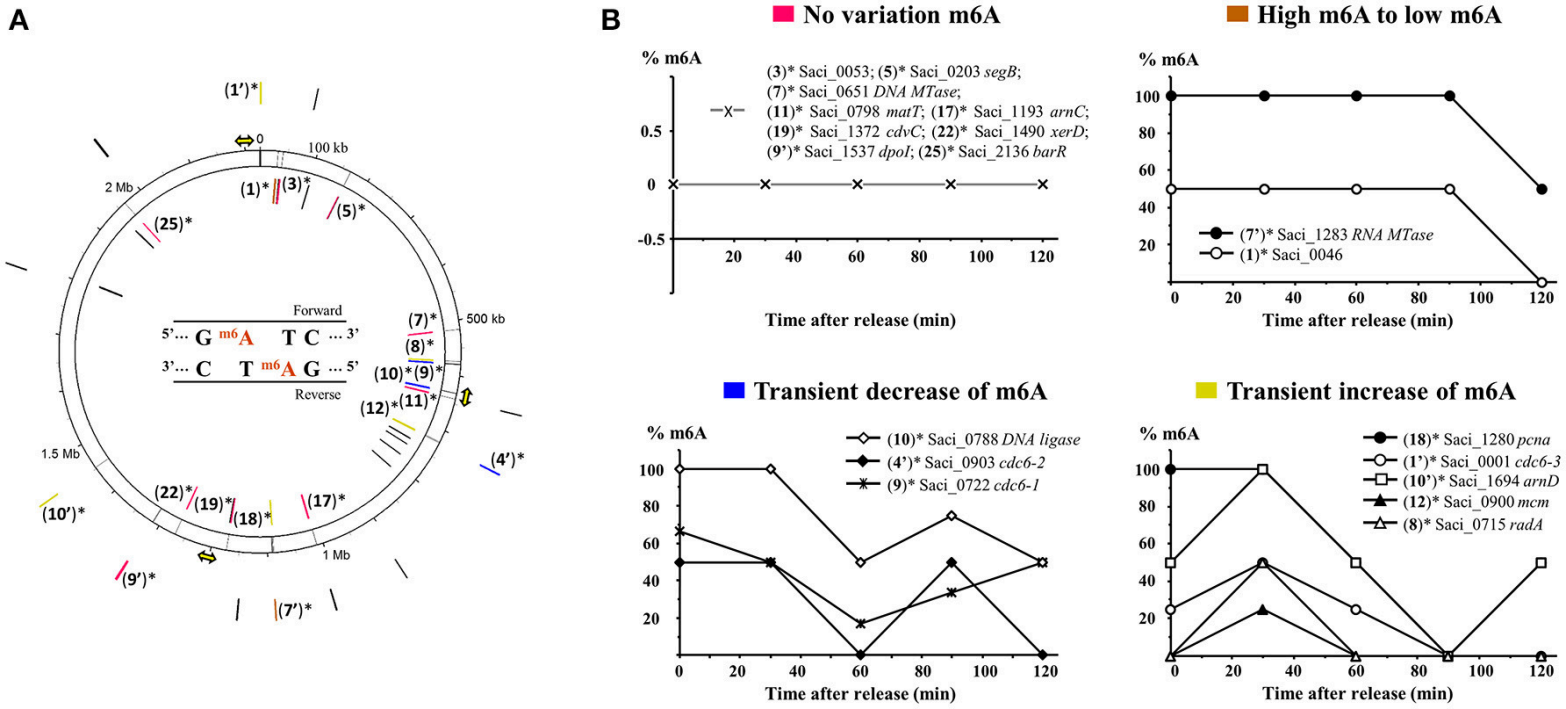
Variations of the percentage of m6A in 37 selected genes during the cell cycle. Distribution of 37 genes along the chromosome of *S. acidocaldarius* represented by the numbered track (black circle) drawn on the Circos plot **(A)**. Gene numbers introduced in Table 4 are used for localizing the corresponding gene along the genome. Genes containing, at least, one 5′-GATC-3′ recognition sequence in two identified contexts, 5′-AGATCC-3′ and 5′-GGATCC-3′, are marked by an asterisk and their recognition sequences are reported in the numbered track by a black line. Four patterns of m6A variations of genes containing 5′-GATC-3′ are identified and associated by a color code **(A,B)**. The three replication origins are represented by yellow arrows.

To further understand the potential role of the m6A modification of the 5′-GATC-3′ core motif in the cell cycle of *S. acidocaldarius*, we decided to analyze the methylation profile of 32 genes whose expression was described as cell-cycle-specific (Lundgren and Bernander, 2007) and 5 genes which were annotated as part of the methylation process (Chen et al., 2005). A list of these 37 genes of interest, clustered and annotated according to their arCOGs and the presence of the 5′-GATC-3′ core motif in their respective nucleic acid sequences is given in Table 4. Most of these genes belonged to the arCOG L (replication, recombination, and repair) and only 19 genes contained at least one 5′-GATC-3′ detected in two contexts 5′-AGATCC-3′ and 5′-GGATCC-3′ (Table 4). All 37 genes were reported along the chromosome of *S. acidocaldarius* and most of them were located closely to replication origins (Figure 4A). Variations of methylation of the adenine in both contexts 5′-AGATCC-3′ and 5′-GGATCC-3′ for genes presenting at least one 5′-GATC-3′ core motif were investigating individually during the cell cycle. Four clusters of genes showing different profiles were obtained (Figure 4B).

**Table 4:** Thirty-seven genes selected and listed according to their arCOG.

**arCOG**	**Genes selected**
**METABOLISM**	
E: Amino acid metabolism and transport	(**11**) Saci_0798^*^
F: Nucleotide metabolism and transport	(**6**′) Saci_1191
**INFORMATION STORAGE AND PROCESSING**	
K: Transcription	(**2**′) Saci_0102; (**3**′) Saci_0800; (**14**) Saci_0942; (**16**) Saci_1012; (**5**′) Saci_1107; (**8**′) Saci_1341; (**25**) Saci_2136^*^
L: Replication, recombination and repair	(**1**′) Saci_0001^*^; (**2**) Saci_0052; (**3**) Saci_0053^*^; (**4**) Saci_0129; (**7**) Saci_0651^*^; (**8**) Saci_0715^*^; (**9**) Saci_0722^*^; (**10**) Saci_0788^*^; (**12**) Saci_0900^*^; (**4**′) Saci_0903^*^; (**15**) Saci_0975; (**18**) Saci_1280^*^; (**7**′) Saci_1283^*^; (**22**) Saci_1490^*^; (**9**′) Saci_1537^*^; (**11**′) Saci_1975; (**12**′) Saci_2156
**CELLULAR PROCESSES AND SIGNALING**	
D: Cell cycle control	(**6**) Saci_0204; (**19**) Saci_1372^*^; (**20**) Saci_1373
T: Signal transduction mechanisms	(**17**) Saci_1193^*^; (**10**′) Saci_1694^*^
V: Defense mechanisms	(**23**) Saci_1989
**POORLY CHARACTERIZED**	
R: General functional prediction only	(**1**) Saci_0046^*^; (**13**) Saci_0925; (**21**) Saci_1374; (**24**) Saci_2119
S: Function unknown	(**5**) Saci_0203^*^

*Genes containing at least one recognition sequence 5′-GATC-3′ in the two contexts (5′-AGATCC-3′ and 5′-GGATCC-3′) are marked by an asterisk. Number into bracket written before the old locus name is used to visualize genes along the genome in Figure 4 on the forward strand (**1**′) or on the reverse strand (**1**)*.

## Discussion

The presence of a restriction-modification system in *S. acidocaldarius* has been well established with the characterization of the endonuclease activity of SuaI (Prangishvili et al., 1985) and the identification of the DNA methylation added on the recognition sequence 5′-GGCC-3′ *via* the DNA MTase M.SuaI (Grogan, 2003). In the current study, we therefore expected to detect this specific DNA methylation on this motif to consider it as an internal control validating the use of the SMRT sequencing technology. The SMRT sequencing confirmed the presence of 570 methylated 5′-GGCC-3′ recognition sequences on the gDNA of *S. acidocaldarius*. This number of recognition sequences is coherent with the number estimated by (Chen et al., 2005), which provides more strength to the SMRT sequencing data. In addition, the SMRT sequencing allowed us to assess the distribution of the 5′-GGCC-3′ motifs along the genome and their methylation state. We found that the majority of recognition sequences were fully methylated independent of the stage of the cell cycle, which is in favor of an involvement of m4C in the discrimination between self and non-self DNA. Furthermore, this result can be interpreted as the presence of hemimethylated recognition sequences after DNA replication should be a short stage to avoid a direct cleavage of this substrate by SuaI and/or a cleavage of unmethylated recognition sequences potentially created after a second round of replication. Therefore, it raises the question of the maintenance of this specific DNA methylation after DNA replication.

Genetic manipulations have demonstrated a need for methylated plasmids before transforming *S. acidocaldarius* cells (Kurosawa and Grogan, 2005; Berkner et al., 2007) which indirectly points to a constitutive expression of the REase SuaI, at least in exponentially growing cells, but nothing is known about the expression of the DNA MTase M.SuaI. However, to explain the constant and high level of m4C, it is tempting to speculate that an induction straight after the action of the replication machinery occurs together with higher affinity of M.SuaI than SuaI for the recognition sequences. The latter proposition is in contradiction with what was observed for other DNA MTases involved in restriction systems since it has been reported that bacterial DNA MTases are less specific than the cognate REases (Jeltsch, 2002). DNA MTases have the ability to slide along the DNA but those involved in restriction-modification systems are described as distributive (Jeltsch, 2002). Therefore it is also reasonable to imagine that the newly transcribed DNA MTase M.SuaI interacts with the DNA replication machinery to act directly on hemimethylated sites and at the same time increasing its activity.

A deletion mutant of SuaI was recently created and characterized (Suzuki and Kurosawa, 2016). This mutant is viable, grows as the wild-type, and allows the uptake of foreign DNA indicating that the endonuclease activity performed by SuaI is mainly dedicated to function in the restriction-modification system. We have shown that the 5′-GGCC-3′ recognition sequences were distributed in genes encoding rRNA and tRNA and in most of the arCOGs except in N (cell motility) and U (Intracellular trafficking and secretion). Altogether, the restriction-modification system SuaI/M.SuaI is important for maintaining the genome integrity of *S. acidocaldarius* by degrading non-self DNA and for protecting genes involved in strategic pathways *via* methylated recognition sequences. Consequently, the absence of recognition sequences in genes belonging to arCOGs N and U might provide less pressure on these genomic areas and allow a certain genome plasticity to adapt to new environmental conditions.

SMRT sequencing combined with dot blot experiments performed in our study revealed the presence of m6A on 5′-GATC-3′ recognition sequences in two contexts for the first time in the *Sulfolobus* genus. This result was not expected since two studies have previously shown by digestion assays that there was no m6A on the 5′-GATC-3′ recognition sequences on gDNA extracted from two *Sulfolobales*—*S. acidocaldarius* and *S. solfataricus* (Barbeyron et al., 1984; Lodwick et al., 1986). In our study, the m6A was not detected in digestion assays, but clearly present in the sequencing data. Indeed, only 19.6% of 5′-GATC-3′ recognition sequences detected by SMRT sequencing were fully methylated which, once cleaved, lead to digestion products undetectable on an agarose gel. Although digestion assay was not sensitive enough to reveal the presence of fully methylated 5′-GATC-3′ recognition sequences on the gDNA of *Sulfolobus*, we have shown that 5′-GATC-3′ motifs were present, at least, in two contexts using the restriction enzyme BamHI in our digestion assay. This result was supported by the SMRT sequencing and we decided to study this methylation independently. Although m6A was detected by SMRT sequencing, the content of methylated adenine calculated on the basis of total adenine on the genome of *S. acidocaldarius* was 0.12%. This percentage can be considered as low when it is compared to what was estimated in some bacteria and eukaryotes (from 0.1 to 2.0% in Ratel et al., 2006), in the range of what was detected by multiple approaches in wild-type *C. elegans* (from 0.01 to 0.4% from Greer et al., 2015) but high if *D. melanogaster* is taken as the reference point (from 0.001 to 0.07% from Zhang et al., 2015). Therefore, the amount of methylated adenine in *S. acidocaldarius* might be considered as low but it is not insignificant, and might be the result of a tight regulation.

In Archaea, the detection of m6A on gDNA of *S. acidocaldarius* is a new discovery, but this DNA methylation has previously been reported in representatives of *Euryarchaeota* (Barbeyron et al., 1984; Lodwick et al., 1986). As a role, this DNA methylation was shown to be part of restriction-modification systems such as PabI/M.PabI in *P. abyssi* and more recently in *Haloferax volcanii* (Watanabe et al., 2006; Ouellette et al., 2015). Thus, we thought that the m6A modification identified on the gDNA of *S. acidocaldarius* could be part of another restriction-modification system. However, if we compare the level of methylation between the well-known m4C and the newly discovered m6A (97 vs. 32.1%, respectively), this hypothesis is no longer valid and this is true for any context of the 5′-GATC-3′ recognition sequences identified by SMRT sequencing. While the number of methylated 5′-GGCC-3′ recognition sequences is widespread along the genome and mostly found fully methylated, fully methylated 5′-GATC-3′ recognition sequences are present in very low amount which seems incompatible with a role of protection of DNA. Therefore, SuaI/M.SuaI, with its constant level of protection throughout the cell cycle, appears to be the only restriction-modification system in *S. acidocaldarius*.

Adenine DNA methylation has mainly been studied in bacteria as well as two well-known solitary adenine DNA MTases, Dam and CcrM. The Dam methylation, corresponding to the addition of a methyl group on adenine on the 5′-GATC-3′ recognition sequence in *E. coli*, has been shown to be involved in a variety of cellular processes including the regulation of DNA replication. The presence of methylated 5′-GATC-3′ recognition sequences *via* Dam at the replication origin was shown to trigger the DNA synthesis (Wion and Casadesus, 2006) which could also be the case for *S. acidocaldarius* since the three origins of replication were estimated to be hypermethylated in asynchronous cells. However, when DNA methylation profiles were investigated during the cell cycle of synchronized cells, a decrease of the number of methylated 5′-GATC-3′ recognition sequences occurred before DNA replication, which is not observed for the Dam methylation. In *C. crescentus*, CcrM methylates the 5′-GANTC-3′ recognition sequences and is involved in the regulation of the cell cycle (Zweiger et al., 1994; Stephens et al., 1996). The study of the dynamics of the adenine methylome during the cell cycle of *C. crescentus* using SMRT sequencing showed that the progressive transition between fully methylated state to hemimethylated state results from the passage of the DNA replication machinery (Kozdon et al., 2013). In our study, the percentage of methylated 5′-GGATCC-3′ recognition sequences in *S. acidocaldarius* was 60.9% in asynchronous cells which could be the result of the DNA replication since most of the cells are actively replicating. However, in synchronous cells, this percentage dropped in G1 instead of in S phase, which should be expected if the DNA replication machinery was triggering a change in the methylation state. When we focused on genes identified to be cell cycle regulated (Lundgren and Bernander, 2007), different profiles could be distinguished but the most surprising was that roughly half of the genes selected did not contain any 5′-GATC-3′ recognition sequences, in the two contexts, within the coding sequence. From this observation, and at least for these genes, we can speculate that the 5′-GATC-3′ is not required to enhance the transcription. It is also known that DNA methylation is involved in transcription regulation at promoter regions by modulating the methylation state of the recognition sequences. This is not only described in mammalian cells with CpG islands and the associated m5C modification (Smith and Meissner, 2013) but also in bacteria with Dam and CcrM (Hale et al., 1998; Reisenauer and Shapiro, 2002; Casadesus and Low, 2006). Therefore, in this study, we searched in the promoter region of 37 selected genes for the presence of the 5′-GATC-3′ recognition sequences, detected in 5′-AGATCC-3′ and/or 5′-GGATCC-3′ contexts by SMRT sequencing, as well as the methylation state (data not shown). None of the investigated promoter regions encompass any of these recognition sequences which indicates the methylation state associated is not part of the regulation, at least not for these genes.

Nevertheless, it has been proposed that m6A affects the DNA double helix modulating the protein-DNA interaction. As a consequence, and due to the low amount of fully methylated 5′-GATC-3′in *S. acidocaldarius*, it is not excluded that the adenine methylation in this microorganism could be involved in signaling one parental strand by recruiting proteins involved in DNA repair and preserve the genetic information.

In this study, we also investigated the presence of m5C on the gDNA extracted from exponential growing *S. acidocaldarius* cells. We were not able to detect this DNA methylation although a recent study revealed this DNA methylation could be found on the 5′-GATC-3′ recognition sequences (Blow et al., 2016). In the latter study, a TET conversion was performed to enhance the detection signal of potential m5C modifications followed by SMRT sequencing (Clark et al., 2013) while, in the present study, we used primary antibodies and SMRT sequencing. These techniques do not exhibit the same sensitivity and the percentage of m5C detected by TET SMRT sequencing is low (10%) which can explain the different results. The presence of this DNA methylation in the genome of a hyperthermophile has been discussed but was not expected since this DNA methylation has a high mutability power and could affect genome stability (Grogan, 2003). However, a gene encoding a potential m5C DNA MTase is annotated in the genome of *S. acidocaldarius* (Chen et al., 2005). Therefore, the presence of this DNA methylation in low amount and at specific stage of the growth could be speculated on. Additionally, it is interesting to notice that the recognition sequences on which m5C occur, according to Blow et al. (2016), are on 5′-RGATCY-3′ (with “R” being a purine and “Y” being a pyrimidine), a motif potentially also modified on the adenine (our study). A cross-talk between these two DNA methylations could be hypothesized and need to be investigated. Since no gene encoding an m6A DNA MTase was annotated in the genome of *S. acidocaldarius* (Chen et al., 2005) and no Dam homologs were identified in *Sulfolobales* (Koike et al., 2005), it is tempting to speculate that M.SuaII might be involved in these two DNA methylations. However, it has been reported that primary sequences and three-dimensional protein structures of DNA cytosine-C5 MTases and DNA N-MTases are distinct from each other (Jeltsch, 2002) which excludes this hypothesis.

Two types of DNA methylations, m4C and m6A, were identified in the current study and we were able to directly map them along the genome. Based on our data, the restriction-modification system SuaI/M.SuaI appears to be the only defense system present in *S. acidocaldarius* and this system might be involved in the plasticity of the genome controlling genetic exchange in certain arCOGs. In addition, sequencing data obtained represents a deep source of information in order to understand the function(s) of adenine DNA methylation in *S. acidocaldarius*. This multiple approaches study, including biochemical characterization, could be extended to other *Sulfolobales* to know whether adenine DNA methylation is conserved through the *Sulfolobus* genus and, finally, get more insight into the role of m6A identify in the regulation of biological pathways.

## Author contributions

A-CL and MC: designed the project; MC: performed the research; A-CL and MC: analyzed the data; A-CL and MC: wrote the paper.

### Conflict of interest statement

The authors declare that the research was conducted in the absence of any commercial or financial relationships that could be construed as a potential conflict of interest.

## Supplementary material

Data sheet 1 Exponentially growing *S. acidocaldarius* cells.

Data sheet 2 Synchronized *S. acidocaldarius* cells at T0 min.

Data sheet 3 Synchronized *S. acidocaldarius* cells at T30 min.

Data sheet 4 Synchronized *S. acidocaldarius* cells at T60 min.

Data sheet 5 Synchronized *S. acidocaldarius* cells at T90 min.

Data sheet 6 Synchronized *S. acidocaldarius* cells at T120 min.
